# Mitochondrial Dysregulation in the Pathogenesis of Diabetes: Potential for Mitochondrial Biogenesis-Mediated Interventions

**DOI:** 10.1155/2012/642038

**Published:** 2011-12-01

**Authors:** Anna-Maria Joseph, Denis R. Joanisse, Richard G. Baillot, David A. Hood

**Affiliations:** ^1^Department of Biology, York University, Toronto, ON, Canada M3J 1P3; ^2^Muscle Health Research Center (MHRC), York University, Toronto, ON, Canada M3J 1P3; ^3^Division of Kinesiology, Laval University, Québec City, QC, Canada G1K 7P4; ^4^Department of Cardiac Surgery, Laval University, Québec City, QC, Canada G1V 4G5; ^5^School of Kinesiology and Health Science, York University, Room 302, Farquharson Life Sciences Building, 4700 Keele Street, Toronto, Ontario, Canada M3J 1P3

## Abstract

Muscle mitochondrial metabolism is a tightly controlled process that involves the coordination of signaling pathways and factors from both the nuclear and mitochondrial genomes. Perhaps the most important pathway regulating metabolism in muscle is mitochondrial biogenesis. In response to physiological stimuli such as exercise, retrograde signaling pathways are activated that allow crosstalk between the nucleus and mitochondria, upregulating hundreds of genes and leading to higher mitochondrial content and increased oxidation of substrates. With type 2 diabetes, these processes can become dysregulated and the ability of the cell to respond to nutrient and energy fluctuations is diminished. This, coupled with reduced mitochondrial content and altered mitochondrial morphology, has been directly linked to the pathogenesis of this disease. In this paper, we will discuss our current understanding of mitochondrial dysregulation in skeletal muscle as it relates to type 2 diabetes, placing particular emphasis on the pathways of mitochondrial biogenesis and mitochondrial dynamics, and the therapeutic value of exercise and other interventions.

## 1. Introduction

Type 2 diabetes is the most common form of diabetes accounting for *∼*90% of diabetic cases and *∼*8% of the total population [1]. Type 2 diabetes is characterized by insulin resistance and is commonly associated with several clinical complications such as hypertension, atherosclerosis, and cardiovascular disease, and these are often collectively referred to as the metabolic syndrome [1]. Although the specific molecular mechanisms underlying type 2 diabetes are not well understood, insulin resistance is believed to result from reductions in glucose transport and phosphorylation and impaired fatty acid metabolism in a number of tissues, notably skeletal muscle [1, 2]. Specifically, defects in this series of reactions are directly associated with increased levels of plasma and intracellular free fatty acids and alterations in insulin signaling pathways [3, 4]. 

Mitochondria have several functions but are most known for their role as key regulators of metabolic activity within the cell by converting energy from the oxidation of macronutrients to ATP. Mitochondrial activity and function in skeletal muscle is a highly controlled process, under the influence of a variety of nuclear and mitochondrial factors that act as metabolic sensors and can adapt to perturbations in cellular nutrient and energy status. The renewal of mitochondria through the process of biogenesis is vital for maintaining mitochondrial integrity, and a diminished capacity for organelle biogenesis has been implicated in the pathogenesis of several diseases such as aging, neurodegeneration, as well as type 2 diabetes [1, 5]. Additionally, muscle mitochondrial metabolism is regulated by a group of morphogenesis machinery proteins which are important for mitochondrial fusion and fission events and also for their independent effects on bioenergetics, programmed cell death, and autophagy [6]. Defects in mitochondrial biogenesis and morphogenesis factors can impair enzyme activity and reduce the oxidative capacity of the cell leading to insufficient oxidation of lipids and increased intramyocellular lipid (IMCL) levels. The inability of mitochondria to utilize these substrates along with their accumulation within muscle has been associated with impaired insulin signaling pathways and reduced glucose uptake [7]. Elevated IMCLs, in association with the increased production of lipid metabolites such as acyl coenzyme A (CoA), diacylglycerol (DAG), ceramides, and reactive oxygen species (ROS) [2, 8], can affect insulin signaling and contribute to insulin resistance associated with type 2 diabetes. Additionally, skeletal muscle from individuals with type 2 diabetes have a higher percentage of type II fibers and a lower percentage of type I fibers when compared to control individuals [9, 10]. Type II fibers have a reduced capacity to oxidize fat [11] and possess unique properties that have been shown to potentiate mitochondrial hydrogen peroxide production and oxidative stress [12]. Therefore, there are likely multiple factors that contribute to the stress environment that intensify the mitochondrial dysregulation observed in type 2 diabetes. 

The multiplicity of mitochondrial functions has made it a logical target for the study of metabolic diseases, and, given that skeletal muscle represents the major site of insulin-stimulated glucose utilization in the body [13, 14], dysregulation of mitochondria is closely associated with insulin resistance and the pathogenesis of type 2 diabetes in muscle. In this paper, we will first discuss key pathways involved in the regulation of mitochondria, with specific attention given to organelle biogenesis, as well as mitochondrial fusion and fission events and their contribution to metabolic perturbations in muscle. In the second part, current therapeutic interventions will be described, with the focus on those related to stimulating mitochondrial biogenesis.

## 2. Mitochondrial Biogenesis

**Figure 1:**
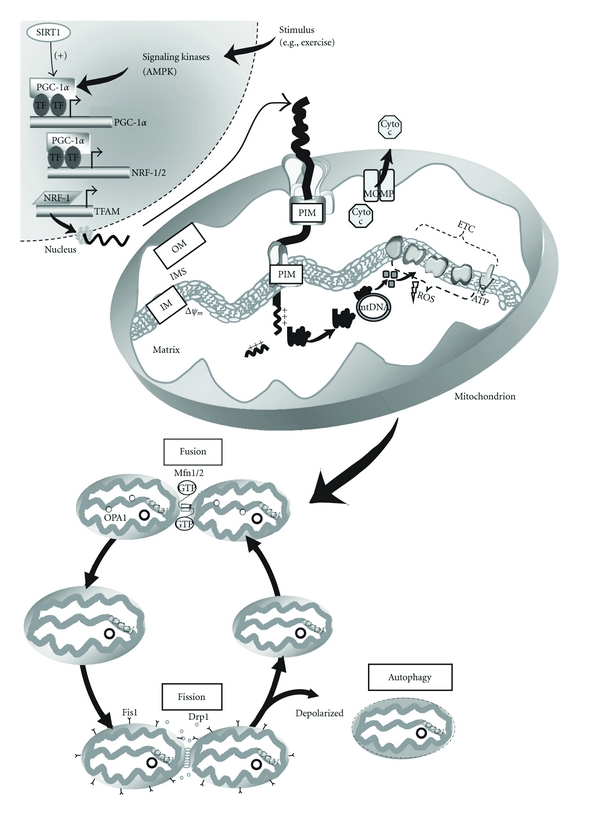
Proposed model of mitochondrial biogenesis. In response to a stimulus such as skeletal muscle contractile activity or exercise, intracellular Ca^2+^ levels, as well as AMP levels, increase leading to the activation of signaling molecules including AMP-activated protein kinase (AMPK). These signaling pathways converge and interact primarily with the transcriptional coactivator peroxisome proliferator-activated receptor-*γ* coactivator-1*α* (PGC-1*α*) which is a master regulator of mitochondrial biogenesis. PGC-1*α* activates its own expression, as well as the expression of the nuclear respiratory factor-1 and 2 (NRF-1/2). Additionally, PGC-1*α* has recently been shown to be deacetylated and activated by the longevity protein sirtuin 1 (SIRT1). NRF-1 and NRF-2 bind and upregulate the expression of nuclear genes encoding mitochondrial proteins (NUGEMPs), as well as the expression of mitochondrial transcription factor A (Tfam). Tfam along with other newly transcribed NUGEMPS are targeted and imported into mitochondrial subcompartments via the protein import machinery (PIM). Within the matrix, Tfam binds to mtDNA and regulates the expression of the 13 mitochondrial DNA (mtDNA) gene products. These proteins are assembled into multisubunit enzyme complexes within the electron transport chain (ETC) and mediate oxidative phosphorylation (OXPHOS) and the production of ATP. Thus, coordinated expression regulated by the two genomes allows for the proper assembly and expansion of the mitochondrial reticulum leading to mitochondrial proliferation and increased mitochondrial number/content. Another important product of the ETC is reactive oxygen species (ROS) that are associated with the mitochondrial membrane potential (Δ*ψ*_m_). Elevated levels of ROS have been shown to activate mitochondrial outer membrane permeabilization (MOMP) and the release of proapoptotic factors such as cytochrome c (Cyt c) into the cytosol that can subsequently activate caspase-dependent signaling cascades leading to mitochondrially-mediated apoptosis. Furthermore, organelle biogenesis requires a continuous cycle of fusion and fission events. Mitochondrial fusion of the outer and inner mitochondrial membranes is mediated by the GTPase proteins, mitofusin 1 and 2 (Mfn1 and Mfn2) and OPA1, respectively. Conversely, mitochondrial fission requires Drp1 and Fis1 which assemble at fission sites on the mitochondrial membrane and induce membrane division. It has been proposed that fission can lead to mitochondria with different Δ*ψ*_m_ and that damaged or depolarized organelles will exit the fusion and fission cycle and will be removed through autophagy.

Skeletal muscle is a malleable tissue and can adapt to alterations in energy status and substrate supply in part via its ability to increase the number of mitochondria. Mitochondrial biogenesis is induced by numerous physiological, environmental, and pharmacological stimuli and results from the transcription and translation of genes both in the nuclear and the mitochondrial genomes [15, 16]. The biogenesis of mitochondria is mediated by changes in many key intracellular events, including transcriptional activation, mRNA stability, posttranslational modification of proteins, and/or alterations in the import and processing of proteins in mitochondria (Figure 1) [17, 18]. These gene products are assembled into functional multisubunit complexes within mitochondria and enhance oxidative capacity and ATP production within the cell. Thus, mitochondria are key regulators of metabolic activity within the cell, and it is these attributes that have made mitochondria a primary focus in the study of metabolic disorders such as type 2 diabetes. 

Some of the early studies examining mitochondrial function and insulin resistance reported reduced mitochondrial content and impaired lipid oxidation in skeletal muscle of obese and type 2 diabetic individuals [19, 20]. The role of mitochondria in obesity and type 2 diabetes has been confirmed by studies examining insulin resistant but otherwise, healthy individuals, with non-insulin-resistant subjects. The insulin-resistant group did not only have higher intramyocellular lipid (IMCL) levels than the control group, but this was associated with a 40% decrease in both oxidative capacity and ATP levels [21]. Impaired mitochondrial oxidative capacity is also an early feature observed in insulin-resistant offspring of individuals with type 2 diabetes [22]. The altered mitochondrial phenotype observed in human skeletal muscle tissue is retained in myocyte cultures obtained from individuals with type 2 diabetes [23]. Furthermore, an A3243G mutation in the tRNA^Leu^ gene in mitochondrial DNA (mtDNA) is the cause of maternally inherited diabetes and deafness (MIDD), and individuals with this mutation will likely develop diabetes as they age [24]. These data support the hypothesis that mitochondrial dysfunction may be one of the early events in the pathogenesis of this disease that predisposes an individual to elevated levels of IMCL, lipid metabolites, and insulin resistance.

In order to elucidate the molecular mechanisms that are responsible for the reduction in mitochondrial content and enzyme activity, earlier studies used DNA microarray techniques to examine the gene expression profile of mitochondrial proteins within skeletal muscle of obese and type 2 diabetic patients. These studies found significant alterations in a wide array of genes responsible for glucose uptake, fatty acid oxidation, and oxidative phosphorylation (OXPHOS) [25, 26]. These include changes in the expression of various nuclear regulatory proteins. Peroxisome proliferator-activated receptor *γ* (PPAR*γ*) coactivator-1 (PGC-1) and their related family members (PGC-1*α*, PGC-1*β*, PRC, and PERC) [27–30] are perhaps among the most well-known regulators of mitochondrial biogenesis. In particular, PGC-1*α* binds and coactivates transcription factors such as the estrogen-related receptor alpha (ERR*α*) and the nuclear respiratory factors 1 and 2 (NRF-1 and NRF-2) to cause the induction of a broad spectrum of genes involved in substrate metabolism and mitochondrial biogenesis. NRF-1 and NRF-2 have been shown to transactivate target genes involved in several mitochondrial processes, including OXPHOS subunits, heme biosynthesis, mitochondrial import machinery, and mtDNA transcription [31–33]. In addition, it has been shown that a dominant negative allele of NRF-1 prevents the ability of PGC-1*α* to induce mitochondrial proliferation, confirming the importance of NRF-1 in PGC-1*α*-driven mitochondrial biogenesis [29]. 

 In skeletal muscle, the importance of PGC-1*α* has been reinforced with data from studies in both cell culture, as well as transgenic mouse models, where PGC-1*α* levels were experimentally altered. Forced expression of PGC-1*α* in cultured muscle cells and cardiac myocytes results in an increase in nuclear and mitochondrial gene expression and mtDNA content [29, 34]. Animals with increased muscle PGC-1*α* have a longer lifespan that is associated with enhanced mitochondrial function, improved insulin sensitivity, and reduced oxidative damage and also show resistance to age-related weight gain [35]. Furthermore, overexpression of PGC-1*α* in mice results in a partial fiber-type transition from white muscle with mostly glycolytic fibers to muscle that appears red and has a high oxidative capacity [36]. This fiber-type conversion coincides with the activation of calcineurin signaling cascades, the coactivation of myocyte-enhancer factor 2 (MEF2) by PGC-1*α*, and the induction of slow gene expression pathways. Calcium- (Ca^2+^-) dependent PGC-1*α* activation was further confirmed in skeletal muscle from transgenic mice overexpressing a constitutively active form of the calcium/calmodulin-dependent protein kinase IV (CaMKIV). These mice displayed increased mtDNA copy number and an upregulation of several enzymes that are involved in fatty acid oxidation and OXPHOS [37]. Additionally, upregulation of PGC-1*α* mRNA and protein with acute and chronic exercise in both animals and humans leads to an increased mitochondrial content through the induction of NRF proteins and mitochondrial transcription factor A (Tfam) [38–40]. 

Regarding metabolic disorders, PGC-1*α* mRNA levels are reduced in certain cohorts of obese and type 2 diabetic individuals [25, 26], and, in some populations, polymorphisms in the PGC-1*α* gene have been linked to a predisposition for type 2 diabetes [41, 42]. PGC-1*α* induces the expression of the insulin-sensitive glucose transporter (GLUT4) by interacting and coactivating the MEF2 transcription regulator [43]. Furthermore, the tissue-specific knockout of Tfam in pancreatic *β* cells leads to the development of diabetes that is associated with a loss of mtDNA and impaired oxidative capacity [44]. Despite these findings, the importance of PGC-1*α* and other mitochondrial regulators of biogenesis in insulin resistance and type 2 diabetes has remained controversial. This is because several studies have shown elevated IMCL levels and reduced mtDNA content in the absence of changes in PGC-1*α* expression (mRNA or protein) or other PGC-1*α*-related target genes [22, 45, 46]. In addition, several studies of muscle-specific PGC-1*α* and/or PGC-1*β* null mice have demonstrated normal glucose tolerance and insulin sensitivity [47, 48]. These studies suggest that alternate mechanisms may also regulate mitochondrial content in metabolic diseases. Clearly more work is required in this area to obtain a better understanding of the molecular pathways mediating insulin sensitivity in both healthy muscle, as well as muscle with metabolic dysfunction. 

Another clue into the molecular function of PGC-1*α* comes with the recent finding that PGC-1*α* is present within mitochondria and specifically localized in a complex with Tfam in mtDNA nucleoids [49]. This surprising finding is also confirmed in animals where, following an acute bout of exercise, PGC-1*α* protein was increased in both the nuclear and mitochondrial subfractions [50]. These preliminary studies suggest that PGC-1*α* coactivates mitochondrial transcription in both the nucleus and mitochondria and indicates the potential of PGC-1*α* as being a central messenger of nuclear-mitochondrial crosstalk during cellular stress. 

Recently, another family of proteins has emerged as crucial regulators of mitochondrial activity and cellular energy metabolism. Sirtuins are a group of class III histone/protein deacetylases that are primarily known for their involvement in promoting lifespan in a number of organisms including yeast, flies, and mice, and they accomplish this by the acetylation and deacetylation of target genes [51]. To date, seven sirtuin mammalian homologs have been identified (SIRT1-7), three of which are mainly localized to mitochondria (SIRT3, SIRT4, SIRT5) [51, 52]. SIRT1 is a NAD-dependent deacetylase that is widely expressed in mammalian cells and activated in response to cellular stress conditions such as with exercise, caloric restriction (CR), and starvation [53–57]. Transgenic mice overexpressing SIRT1 have similar physiological and behavioral phenotypes as calorie restricted mice. These animals have less body fat, are more metabolically active, and display improved insulin sensitivity and glucose tolerance [58]. The CR-mediated phenotype is dependent on SIRT1, since knockout of this gene diminishes these adaptations [59]. Studies investigating the role of SIRT1 in diabetes and skeletal muscle are limited. In cardiac muscle of Otsuka Long-Evans Tokushima fatty (OLETF) rats, SIRT1 levels were lower in these animals than in control rats. Treatment with pioglitazone, a PPAR*γ* agonist, enhanced SIRT1 expression [60]. Similar attenuations in SIRT1 levels were reported in adipocytes from ob/ob mice [61] and adipose tissue from obese women [62]. SIRT1 gain-of-function studies in various models of insulin resistance and diabetes have revealed improved glucose tolerance and decreased energy expenditure that is due to lower hepatic glucose production and increased adiponectin levels [63] suggesting that SIRT1-mediated longevity may be related to improvements in insulin sensitivity. 

SIRT1 regulates AMP activated-protein kinase (AMPK), a key energy-sensing molecule that is activated during conditions of low ATP and high AMP (increased AMP/ATP ratio) such as with exercise [64] and CR [65]. Interestingly, both SIRT1 and AMPK together or independently activate PGC-1*α* to induce the expression of genes involved in glucose and fatty acid metabolism and restore ATP levels [66–69]. AMPK activity levels decline with aging, and this is associated with reduced insulin sensitivity and diminished fatty acid oxidation, suggesting that AMPK is an important regulator of mitochondrial metabolism in muscle [70, 71].

More recently, another histone/protein deacetylase has gained increasing attention as a key regulator of metabolic activity in muscle. SIRT3 contains a cleavable N-terminal mitochondrial targeting signal that permits its import into mitochondrial subcompartments [51]. SIRT3 levels were diminished with aging and a high fat diet [65, 72] and increased in response to exercise, CR, and fasting [65, 72, 73]. It is likely that, similar to SIRT1, SIRT3 is a key regulator of muscle adaptation since it also targets PGC-1*α* and influences mitochondrial transcriptional regulation in muscle [65, 74]. 

It is important to remember that skeletal muscle contains two subpopulations of mitochondria, subsarcolemmal (SS) and intermyofibrillar (IMF) mitochondria, that display key differences in biochemical and functional properties [75]. These differences are crucial because it has been proposed that, due to their close proximity to myonuclei, SS mitochondria may be important for driving processes at the cell surface, including the propagation of insulin signaling pathways, fatty acid oxidation, and glucose transport [75, 76]. In contrast, IMF mitochondria are thought to provide energy for muscle contractions [75]. In both SS and IMF subfractions, electron transport chain (ETC) activity was reduced in obese and type 2 diabetic subjects when compared to lean subjects [77]. Interestingly, the decrement in enzyme activity was more pronounced in the SS, compared to the IMF subfraction, suggesting that SS mitochondria may be more readily affected in states of altered glucose homeostasis. These data are consistent with previous findings showing that SS mitochondria are more labile in response to metabolic changes [78, 79].

## 3. Mitochondrial Fusion and Fission

The adaptability of skeletal muscle is also associated with mitochondrial morphological plasticity. Mitochondria are dynamic and readily adapt to changes in cellular energy demands through network remodeling and continuous fusion and fission [5, 80]. Under normal conditions, mitochondrial fusion results in the formation of an interconnected mitochondrial network that allows the mixing and redistribution of proteins and mtDNA and which has been hypothesized to prevent the accumulation of mutated or damaged mtDNA in a cell [5, 81, 82]. In contrast, mitochondrial fission leads to mitochondria with a fragmented morphology facilitating the segregation of damaged mitochondria that can then be targeted for degradation via autophagy [5]. Although the molecular mechanisms mediating these morphological changes remain largely unknown, recent studies have identified distinct fusion and fission machinery that appear to regulate these processes [5, 81, 82]. 

The fusion of mitochondria in mammalian cells is mediated by several proteins, the most well known being the nuclear-encoded dynamin-related guanosine triphosphatases (GTPase), mitofusin 1 (Mfn1), and mitofusin 2 (Mfn2). Although these mitofusins share greater than 70% homology, they have different GTPase activity levels and display distinct expression patterns, with Mfn2 present in higher amounts in tissues such as heart and skeletal muscle [83, 84]. Additionally, mutations in Mfn2 cause Charcot-Marie-Tooth (CMT) disease-type 2A, the most common form of CMT disease and an inherited neuropathy leading to progressive weakness and sensory loss [85, 86]. While mitofusins are responsible for tethering and fusion of the outer mitochondrial membrane, another dynamin family GTPase, Optic Atrophy 1 (OPA1), is required for inner membrane fusion. OPA1 was first identified through its involvement in the neurodegenerative disease known as autosomal dominant optic atrophy (ADOA) [87]. Alternative splicing of OPA1 produces multiple variants that are distinctly present in different species and tissues [88]. Furthermore, posttranslational modification of OPA1 by mitochondrial processing peptidase (MPP) results in different length isoforms that vary based on their localization and function [89, 90]. Muscle-specific Mfn1 and Mfn2 knockout mice with a diminished capacity for mitochondrial fusion have impaired mitochondrial function and a loss of muscle mass that are associated with increased mtDNA point mutations and deletions and severe mtDNA depletion [91]. Similarly, silencing of OPA1 in mammalian cells blocked mitochondrial fusion and resulted in mitochondrial fragmentation, decreased OXPHOS, poor cell growth, and reduced mitochondrial membrane potential (Δ*ψ*_m_) [92]. These data indicate that mitochondrial fusion is important for maintaining the integrity of the organelle by allowing the intramitochondrial exchange of damaged mitochondria, preventing their localization within specific organelles and their accumulation within the cell. 

Mitochondrial homeostasis is also regulated by fission machinery such as dynamin-related protein 1 (DLP1/Drp1) and fission protein 1 (Fis1). Drp1 is a dynamin-related GTPase that is found in the cytosol and recruited by Fis1 to scission sites on the mitochondrial outer membrane to induce mitochondrial fission. Downregulation of Drp1 in HeLa cells leads to a loss in mtDNA, reduced mitochondrial respiration, and higher levels of ROS, all of which are associated with mitochondrial dysfunction [93]. Also, blocking fission by reducing the levels of Drp1 and Fis1 genes in a human cell line with a mtDNA mutation exacerbates the abundance of mutant mtDNA compared with wild-type mtDNA [94]. These studies imply that mitochondrial fusion and fission are involved in mtDNA quantity and quality control and are required for the maintenance of healthy organelles. 

It is also becoming more and more apparent that mitochondrial morphology is directly linked to mitochondrial function and substrate utilization. Cells with a high mitochondrial fusion capacity display interconnected mitochondria associated with increased OXPHOS, while cells with high mitochondrial fission have fragmented mitochondria and rely more on anaerobic metabolism pathways for energy [81]. A hallmark of mitochondrial biogenesis is an expansion of the mitochondrial reticulum, thereby allowing the propagation of signaling pathways and the mixing of metabolites. A perfect example of this adaptation in muscle occurs with exercise, whereby repeated bouts of an exercise stimulus lead to muscle adaptations. Substantial evidence in the last decade has shown the involvement of mitochondrial network remodeling in these exercise-induced adaptations. The expression levels of Mfn1, Mfn2, and Fis1 have been shown to be increased in skeletal muscle following an acute bout of exercise in both animals and humans [95, 96]. Additionally, transcript levels of mitochondrial dynamics proteins are upregulated with endurance exercise in healthy subjects, and these are closely correlated with muscle OXPHOS activity and PGC-1*α* mRNA content [97]. An important finding of these studies is that fusion and fission machinery respond rapidly to an exercise stimulus and that the levels of these proteins are dependent on the type of exercise [95, 96]. 

**Figure 2:**
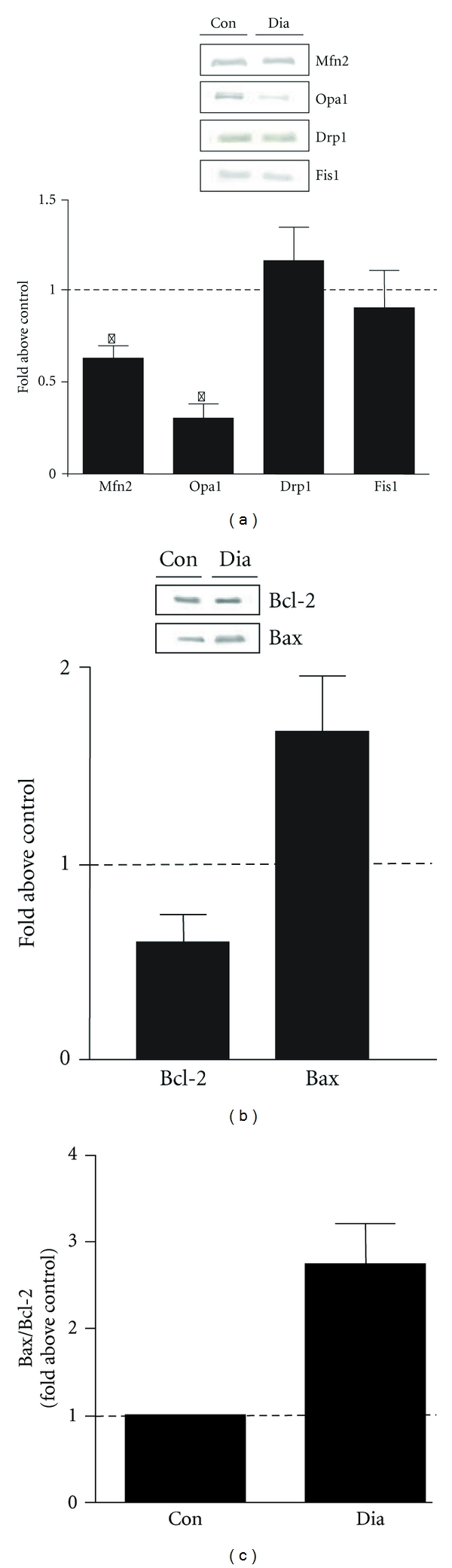
(a) Mitochondrial morphology proteins in type 2 diabetes. The research volunteers that participated in this study were obese subjects with type 2 diabetes undergoing coronary bypass surgery and were all male between 48 and 75 years of age. Biopsies from the vastus medialis muscle were removed from both control and type 2 diabetic subjects from within incisions of the inner thigh and protein analyses performed. The protocol was approved by the Medical Ethics Committees of Laval University and Laval Hospital, and all subjects provided informed written consent. Representative western blots of fusion proteins Mfn2 and OPA1 and fission proteins Drp1 and Fis1 from the vastus medialis muscle of control (Con) and type 2 diabetic subjects (Dia). A summary of repeated experiments is shown below with values expressed as a fold over control. Values are means ± SE; *n* = 4–9; *P < 0.05 versus Con. (b) Indicators of apoptotic susceptibility in type 2 diabetes. Western blots of the antiapoptotic protein Bcl-2 and the proapoptotic protein Bax in vastus medialis muscle of Con and Dia individuals and the graphical representation of the data is shown below. Values are means ± SE; *n* = 4–9. (c) The ratio of Bax/Bcl-2 in Dia subjects when compared to Con.

Given the involvement of mitochondrial dynamics in muscle metabolism, perturbations in the fusion-fission balance caused by changes in the levels of the molecular machinery have been shown to lead to abnormal mitochondrial morphology and to negatively impact mitochondrial function. Mitochondrial gene expression profiles and function are associated with changes in overall mitochondrial morphology in skeletal muscle from diabetic rats [83], as well as in humans with type 2 diabetes [83, 98]. In particular, mitochondria in skeletal muscle from type 2 diabetics are smaller in size than mitochondria present in lean subjects, and they also contain abnormal cristae structure that would suggest defects in the inner membrane [83, 98, 99]. In our laboratory, we have found reduced levels of fusion proteins Mfn2 and Opa1, but no alterations in fission proteins Drp1 or Fis1 in skeletal muscle from type 2 diabetic individuals (Figure 2(a)). Similar reports of decreased Mfn2 expression and aberrant mitochondrial morphology have been documented by other studies in type 2 diabetics [98, 99].

Loss of function of the fusion protein Mfn2 leads to impaired mitochondrial metabolism and is associated with reduced mitochondrial oxygen consumption, membrane potential, and glucose metabolism in a variety of tissues [83, 92, 100]. In contrast, Mfn2 gain of function increases substrate oxidation and improves mitochondrial metabolism in HeLa cells [100]. Additionally, similar to other key metabolic factors, Mfn1 and Mfn2 are increased in response to weight loss and exercise in healthy, obese, and type 2 diabetic individuals [95, 101] and are increased also in mice subject to CR in mice [102]. This is dependent primarily on PGC-1*α* and ERR*α* transactivation of the Mfn2 promoter, suggesting that fusion is an important signaling event for mitochondrial biogenesis and healthy insulin signaling in muscle [95, 103]. Additionally, reduced levels of OPA1 are associated with insulin resistance in several cell types [104], as well as in human fibroblasts from patients with ADOA with impaired OXPHOS and reduced mitochondrial fusion events [105]. These findings are in keeping with the observation that mitochondrial fusion is an important signaling event for mitochondrial biogenesis in muscle. For the most part, the regulatory mechanisms and the functional importance of these protein changes in obesity and type 2 diabetes remain enigmatic, and more work is required to elucidate the regulatory factors of the other fusion and fission machineries. 

### 3.1. Mitochondrial Dynamics and Cell Death

The finding that aberrant mitochondrial morphology is present in cells undergoing apoptosis has led to the examination of the possible link between mitochondrial fusion and fission processes and programmed cell death. A hallmark of apoptosis is mitochondrial outer membrane permeabilization (MOMP) and the release of cytochrome c that activates proapoptotic signaling cascades. This results in the fragmentation of DNA [106]. Imaging experiments have demonstrated that mitochondrial fragmentation occurs concurrently with MOMP. This is primarily due to fission, since either the inhibition of fission proteins or upregulation of fusion proteins can delay or prevent mitochondrial fragmentation, along with the induction of proapoptotic signaling events including MOMP, cytochrome c release, and cell death [89, 107]. 

Studies conducted to elucidate the molecular mechanisms responsible for the association between mitochondrial morphology and apoptosis revealed a physical interaction between morphogenesis proteins and the apoptosis machinery. In particular, proapoptotic proteins such as Bax and Bak interact with Mfn proteins [108, 109], and similar findings were reported between the fission machinery and the Bcl-2 family of proteins [110, 111]. It is highly likely that the physical interaction between apoptotic proteins and mitochondrial morphogenesis proteins is another means of regulating cell death. However, not all studies are consistent with this theory, and more work is required to clearly show whether mitochondrial fusion and fission are an important part of the apoptosis signaling cascade. Nonetheless, altering levels of fusion and fission events can confer protection to the cell, and this introduces a potential mechanism of decreasing apoptotic susceptibility through the modulation of the fusion and fission machinery. 

Mitochondria represent the major source of ROS production within the cell and increased levels of ROS are a likely culprit in a variety of pathophysiological conditions, including type 2 diabetes. Increased ROS production was associated with altered mitochondrial morphology in myotubes cultured in high glucose conditions, as well as in diet-induced diabetic mice [112]. Furthermore, increased oxidative stress within mitochondria arising from impaired oxidative metabolism may contribute to greater lipid peroxidation and damage to cell membranes and DNA, activating a cascade of signaling events that further exacerbate the severity of the disease [113]. A number of studies have found that altering mitochondrial fusion and fission events can influence ROS production in mitochondria. For instance, increasing mitochondrial fusion or decreasing mitochondrial fission during hyperglycemia can prevent mitochondrial fragmentation and reduce ROS levels in the cell [107]. Treatment of cells with hydrogen peroxide, antimycin A, or rotenone to increase ROS production resulted in fragmented mitochondria [107, 114], while addition of an antioxidant reduced ROS levels and inhibited mitochondrial fragmentation [115]. The effect of mitochondrial fusion and fission machinery on apoptosis is likely associated with its ability to modulate ROS levels. Thus, mitochondrial dynamics is a relatively new field in mitochondrial biology, and despite growing progress, many questions still remain regarding how alterations in mitochondrial shape affect mitochondrial bioenergetics, morphology, and ROS production, as well as the sequential order of these events in skeletal muscle with obesity and type 2 diabetes.

Furthermore, a growing area of interest in the field of metabolic diseases is the study of obesity in the elderly, the prevalence of which has considerably increased in the last few years [116, 117]. As we age, there is a progressive decline in muscle mass and strength, as well as an increased fat mass. This combination of sarcopenia and obesity has recently been defined as “sarcopenic obesity” [116, 117]. While the molecular mechanisms mediating a loss of muscle mass in obesity and type 2 diabetes are unknown, several theories, including increased apoptosis, have been brought forward. Recently, Sishi et al. [118] have demonstrated that, as a result of diet-induced obesity, skeletal muscle of adult rats displayed muscle atrophy along with increased apoptosis. This was evident by increased levels of caspase-3 and poly(ADP-ribose) polymerase (PARP), two hallmark features of cell death. Additionally, electron micrographs of muscle fibers from obese and type 2 diabetic individuals display an altered mitochondrial structure and fragmented mitochondria, indicating the potential involvement of apoptosis [99]. We have obtained similar results of increased apoptotic signaling, with a trend toward higher levels of the proapoptotic protein Bax and lower levels of the antiapoptotic protein Bcl-2 observed in muscle from type 2 diabetics (Figure 2(b)). This indicates a greater Bax/Bcl-2 ratio (Figure 2(c)) and suggests that muscle from type 2 diabetics has a greater susceptibility to apoptosis when compared to healthy individuals. Although these studies point to the involvement of apoptosis in sarcopenic obesity, much more research is required to characterize apoptotic signaling pathways in obesity and type 2 diabetes.

### 3.2. Mitochondrial Dynamics and Autophagy

Mitochondrial dynamics are also involved in another form of cell death known as autophagy. Autophagy protects the cell through the selective degradation and recycling of organelles such as mitochondria by lysosomal machinery [119, 120]. Under normal conditions, autophagy prevents the accumulation of damaged mitochondria within the cell that can trigger apoptotic pathways and irreversible cell death. Autophagy is particularly important for postmitotic tissues such as skeletal muscles that are exposed to high levels of oxidative stress and that do not have an inherently high capacity for regeneration [119, 120]. 

Recent studies have shed light on the function and regulation of autophagic pathways in skeletal muscle and have implicated the involvement of mitochondrial fission. The monitoring of individual mitochondria in cultured cells has revealed that daughter mitochondria generated by mitochondrial fission display different mitochondrial membrane potentials (Δ*ψ*_m_), with one mitochondrion possessing a high Δ*ψ*_m_ and the other a low Δ*ψ*_m_. Interestingly enough, depolarized mitochondria with a lower Δ*ψ*_m_ were selectively degraded by autophagy and displayed a lower fusion capacity when compared to mitochondria with a high Δ*ψ*_m_. The reduced capacity for fusion in this subpopulation of mitochondria was attributed to low levels of the OPA1 protein [104]. Increased autophagy levels have also been reported in Fis1-overexpressing cells, indicating the potential for both fusion and fission events in regulating autophagy signaling pathways and mitochondrial turnover [121].

Reduced autophagy has been reported in several tissues with age [122–124], and this is associated with the presence of enlarged mitochondria. In a recent study conducted by Masiero and Sandri [125], muscle-specific knockout of the autophagy-related protein 7 (Atg7) resulted in muscle loss and weakness associated with abnormal mitochondria and the accumulation of protein aggregates and vacuoles. Impaired glucose homeostasis and abnormal mitochondrial structure were also observed in Atg7-deficient pancreatic *β* cells [126]. Furthermore, electron micrographs of muscle fibers from obese and type 2 diabetic subjects revealed that mitochondria are smaller and contain abnormal inner membrane structure when compared to muscle form lean subjects [83, 98]. Additionally, the presence of vacuole-like structures which, when stained with a membrane marker, contain fragmented mitochondria could be detected [99]. Whether these vacuoles represent autophagosomes engulfing damaged or energy-deficient mitochondria still requires confirmation. These studies suggest that the clearance of damaged proteins and organelles such as mitochondria is vital for maintaining cellular integrity. Whether the dysregulation of autophagy contributes to the pathogenesis of type 2 diabetes remains to be investigated.

## 4. Mitochondrial-Mediated Therapeutic Interventions

As mentioned above, factors involved in mitochondrial biogenesis are vital for tissue-specific metabolic control, and recent research has focused on modalities that can improve mitochondrial function and substrate oxidation by stimulating mitochondrial biogenesis and nutrient-sensing pathways. These include physiological and macronutrient interventions, as well as pharmacological interventions. 

### 4.1. Physiological and Macronutrient Interventions

#### 4.1.1. Exercise and Weight Loss

Skeletal muscle from obese and type 2 diabetic individuals is characterized by an impaired oxidative capacity and increased IMCL content [127, 128]. Recent studies to identify potential therapeutic modalities in obese/type 2 diabetic individuals have shown the effectiveness of both acute and chronic exercise to increase muscle glucose disposal, fatty acid oxidation, and mitochondrial biogenesis [129–131]. These changes are mediated by key metabolic factors such as AMPK and SIRT1 that are stimulated with reduced energy states and directly activate the PGC-1*α*-mediated induction of target genes, including NRF-1, Tfam, and Mfn2, as well as genes involved in glucose and fatty acid oxidation [69, 95, 96, 130]. Obese and type 2 diabetic individuals have impaired activation of these signaling pathways, and their responses are attenuated when compared to healthy subjects suggesting that a higher intensity and/or duration of exercise is required to achieve the same adaptations in these patients [130, 132]. 

Weight loss alone is associated with a reduction in IMCL content [20, 133]. However, the beneficial effects of exercise on insulin resistance occur in the absence of a change in lipid content and active lipid intermediates (e.g., ceramides, DAGs, CoAs) and in some cases even elevated IMCL levels [134, 135]. In a study conducted by He et al. [136] using a combination of weight loss and moderate-intensity exercise, increased insulin sensitivity was associated with a reduction in the size of the lipid droplets within skeletal muscle and not the total amount of lipid. The reduced size of the lipid droplets was linked to an improved aerobic capacity, suggesting that increased oxidative enzyme activity resulting from physical activity allowed for the more efficient oxidation of lipids. This is consistent with studies of trained athletes that have been reported to have higher IMCL levels, coinciding with greater oxidative enzyme capacity and improved insulin sensitivity [137, 138]. However, the adaptations that occur in patients with obesity and type 2 diabetes following these interventions do not result in an increased mtDNA content that is typical of mitochondrial biogenesis in healthy muscle. In addition, mitochondria from these individuals display abnormal morphology with altered inner membrane cristae structure [45]. These results are in keeping with the observation that the induction of mitochondrial biogenesis in patients with this metabolic condition differs from the molecular processes typically observed in healthy individuals.

#### 4.1.2. Macronutrient Modulation

The beneficial effects of nutritional interventions on health and lifespan have been known for decades. Nutrient deprivation by way of CR (20–40% reduction) increases lifespan in a number of species ranging from insects to mammals [139, 140]. The efficacy of CR in muscle is mediated by a variety of cellular and molecular changes including increased mitochondrial biogenesis and reduced metabolic rate and ROS levels, as well as increased mitochondrial autophagy [141–144]. Additionally, CR alters substrate utilization in muscle from carbohydrate metabolism towards a greater fatty acid oxidation [145]. The effects of CR have also been reported in humans albeit to a lesser extent than in animals. Civitarese et al. showed that 6 months of 25% CR in healthy young individuals resulted in increased mitochondrial biogenesis that was associated with higher levels of SIRT1, PGC-1*α*, and Tfam protein. Skeletal muscle from these subjects displayed increased mtDNA content along with decreased levels of DNA damage [146]. CR (25%) has also been shown to improve insulin sensitivity and increase mitochondrial density and oxidative enzyme activity in skeletal muscle from type 2 diabetic individuals [131]. Furthermore, prolonged CR in obese individuals with type 2 diabetes decreased myocardial triglyceride content that was associated with improved myocardial function [147]. Thus, CR may be an effective way to increase mitochondrial biogenesis in a wide range of species and tissues; however, more studies are required to characterize its role in insulin signaling in muscle from type 2 diabetics.

#### 4.1.3. Pharmacological Stimulation of Mitochondrial Biogenesis

In an effort to reduce the severity of insulin resistance and the clinical phenotype associated with type 2 diabetes, different classes of drugs have been developed that stimulate/induce mitochondrial biogenesis and morphogenesis. Peroxisome proliferator-activated receptors (PPARs) are a family of nuclear hormone receptors that mediate the expression of a wide array of genes involved in glucose and fat metabolism. Modulation of the PPAR-activated pathway may have therapeutic potential for metabolic disorders. For example, thiazolidinediones (TZDs), such as rosiglitazone, troglitazone, and pioglitazone, are classified as PPAR*γ* agonists that improve insulin sensitivity by increasing substrate metabolism and reducing FFA levels and ROS production [148, 149]. The PPAR agonist bezafibrate has been shown to increase mitochondrial enzyme activity, as well as the levels of several respiratory chain subunit proteins in human fibroblasts with respiratory chain deficiency [150]. 

Another class of drugs gaining wide acceptance in the treatment of diabetes are sirtuin 1 (SIRT1) activators. Transgenic mice overexpressing SIRT1 display lower whole-body energy requirements and have decreased rates of oxygen consumption resulting in higher metabolic efficiency when compared to their wild-type littermates. Additionally, these animals display decreased susceptibility to the development of diabetes [63]. The therapeutic potential of resveratrol, a polyphenolic extract from grape skins and red wine, has been gaining increasing popularity as a CR mimetic due to its ability to induce mitochondrial biogenesis via SIRT1-mediated activation of PGC-1*α* in mice. Furthermore, these mice have improved insulin sensitivity and are refractory to high-fat diet-induced obesity [151, 152]. Small SIRT1 activator molecules appear to mimic the effect of CR by altering the expression of target genes involved in substrate metabolism and antioxidant defenses thereby improving metabolic function [151, 152]. In a recent study by Milne et al. [153], obese animals treated with small SIRT1 activator molecules were shown to display enhanced mitochondrial function and improved insulin sensitivity, indicating the potential use of these drugs in the treatment of type 2 diabetes. 

Another group of mitochondrial biogenesis-stimulating agents showing therapeutic promise are AMPK activators such as 5-aminoimidazole-4-carboxamide-1-*β*-d-ribofuranoside (AICAR) and metformin. The addition of AICAR to C_2_C_12_ myoblasts upregulated PGC-1*α* gene expression through SIRT1 [154], at least in part via transcriptional activation [155]. The administration of AICAR or metformin improved insulin resistance and delayed the onset of diabetes [156, 157]. Furthermore, metformin has been shown to improve insulin sensitivity and has been consistently used as an antidiabetic agent [158]. Thus, AMPK activators have shown great promise as a pharmacological intervention for type 2 diabetes, and future studies in this area will further our understanding of AMPK signaling pathways and promote the development of additional potentially effective antidiabetic agents.

## 5. Concluding Remarks

**Figure 3:**
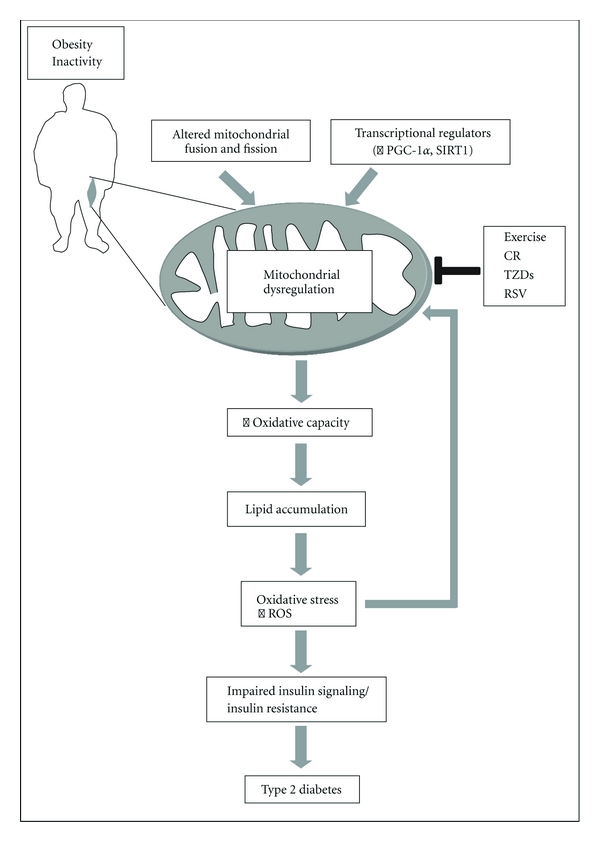
Simplified scheme that illustrates the role of mitochondrial dysregulation in the pathogenesis of type 2 diabetes in skeletal muscle. Obesity and physical inactivity can result in mitochondrial dysregulation through alterations in crucial transcriptional activators (e.g., PGC-1*α* and SIRT1), as well as impaired fusion and fission leading to aberrant mitochondrial morphology. These changes can subsequently lead to reduced oxidative capacity and cause lipid metabolite accumulation, increased oxidative stress, and the production of reactive oxygen species (ROS). Over time, the accumulation of ROS can damage DNA, proteins, and lipids, further exacerbating mitochondrial dysfunction. Collectively, these factors contribute to impaired insulin signaling pathways and increase the risk of type 2 diabetes. On the other hand, physiological interventions, including exercise and caloric restriction (CR), as well as pharmacological agents such as thiazolidinediones (TZDs) and resveratrol (RSV) have been shown to stimulate mitochondrial biogenesis and reduce mitochondrial dysfunction that is observed with type 2 diabetes in muscle.

It is well established that mitochondria are intricately involved in the pathogenesis of type 2 diabetes. This is because of the instrumental role that mitochondria play in lipid and carbohydrate metabolism, their morphological and functional plasticity in response to inactivity and disease, and their involvement in apoptosis and autophagy. Whether the alterations observed in mitochondrial function are a cause, or a consequence, of insulin resistance and type 2 diabetes remains under debate. Despite the fact that there is conflicting evidence regarding the extent of mitochondrial dysfunction in type 2 diabetics, there is a consensus among studies that skeletal muscle mitochondria from diabetic animals or humans exhibit impairments in key transcriptional regulators. In addition, alterations in genes involved in mitochondrial fusion and fission, as well as aberrant mitochondrial morphology, are commonly associated with the increased production of ROS and the accumulation of damaged DNA, proteins, and lipids. Collectively, these transcriptional alterations can impair insulin signaling pathways leading to insulin resistance and the development of type 2 diabetes (Figure 3). The potential to stimulate mitochondrial biogenesis and morphogenesis through physiological interventions such as exercise, CR, or pharmacological mimetics of mitochondrial biogenesis has shown great promise in improving insulin sensitivity and attenuating the clinical phenotype associated with these metabolic disorders. Thus, the closer we come to understanding the molecular mechanisms governing these pathways during normal physiological conditions, the better chance we have to develop new therapeutic strategies that may one day prevent or treat type 2 diabetes.
